# Regional changes in brain metabolism during the progression of mild cognitive impairment: a longitudinal study based on radiomics

**DOI:** 10.1186/s41824-024-00206-8

**Published:** 2024-07-01

**Authors:** Xuxu Mu, Caozhe Cui, Jue Liao, Zhifang Wu, Lingzhi Hu

**Affiliations:** 1 Shanxi Key Laboratory of Molecular Imaging, Shanxi Medical University, Taiyuan, 030001 Shanxi People’s Republic of China; 2 Department of Nuclear Medicine, First Hospital of Shanxi Medical University, Shanxi Medical University, Taiyuan, 030001 Shanxi People’s Republic of China; 3 Collaborative Innovation Center for Molecular Imaging of Precision Medicine, Shanxi Medical University, Taiyuan, 030001 Shanxi People’s Republic of China

**Keywords:** Alzheimer's disease, Machine learning, Positron emission tomography, Mild cognitive impairment, Radiomics

## Abstract

**Background:**

This study aimed to establish radiomics models based on positron emission tomography (PET) images to longitudinally predict transition from mild cognitive impairment (MCI) to Alzheimer's disease (AD).

**Methods:**

In our study, 278 MCI patients from the ADNI database were analyzed, where 60 transitioned to AD (pMCI) and 218 remained stable (sMCI) over 48 months. Patients were divided into a training set (n = 222) and a validation set (n = 56). We first employed voxel-based analysis of 18F-FDG PET images to identify brain regions that present significant SUV difference between pMCI and sMCI groups. Radiomic features were extracted from these regions, key features were selected, and predictive models were developed for individual and combined brain regions. The models' effectiveness was evaluated using metrics like AUC to determine the most accurate predictive model for MCI progression.

**Results:**

Voxel-based analysis revealed four brain regions implicated in the progression from MCI to AD. These include ROI1 within the Temporal lobe, ROI2 and ROI3 in the Thalamus, and ROI4 in the Limbic system. Among the predictive models developed for these individual regions, the model utilizing ROI4 demonstrated superior predictive accuracy. In the training set, the AUC for the ROI4 model was 0.803 (95% CI 0.736, 0.865), and in the validation set, it achieved an AUC of 0.733 (95% CI 0.559, 0.893). Conversely, the model based on ROI3 showed the lowest performance, with an AUC of 0.75 (95% CI 0.685, 0.809). Notably, the comprehensive model encompassing all identified regions (ROI total) outperformed the single-region models, achieving an AUC of 0.884 (95% CI 0.845, 0.921) in the training set and 0.816 (95% CI 0.705, 0.909) in the validation set, indicating significantly enhanced predictive capability for MCI progression to AD.

**Conclusion:**

Our findings underscore the Limbic system as the brain region most closely associated with the progression from MCI to AD. Importantly, our study demonstrates that a PET brain radiomics model encompassing multiple brain regions (ROI total) significantly outperforms models based on single brain regions. This comprehensive approach more accurately identifies MCI patients at high risk of progressing to AD, offering valuable insights for non-invasive diagnostics and facilitating early and timely interventions in clinical settings.

**Supplementary Information:**

The online version contains supplementary material available at 10.1186/s41824-024-00206-8.

## Introduction

Mild cognitive impairment (MCI) is a cognitive state that lies between normal aging and Alzheimer's disease (AD), brought about by various neurological disorders. Clinically and psychologically, MCI is defined as a measurable cognitive deficit in at least one domain, in the absence of dementia or impairment of daily life activities (Castellani et al. 2010). In clinical practice, over time, some patients with MCI remain stable (sMCI), and a few even regain normal function (Fatemi et al. 2022). However, others progress to AD (pMCI). Studies show that the annual probability of patients with MCI transitioning to AD is 10–15%, approximately four times the conversion rate of the healthy control group. Therefore, early intervention in MCI patients with a high-risk profile may effectively delay disease progression (Chan et al. 2021).

Common diagnostic and predictive methods for AD in clinical settings include measurements based on biochemical indicators, such as cerebrospinal fluid tau protein and Amyloidβ-42 deposition, and cognitive ability scale tests like MMSE (Mini-Mental State Examination) and CDRSB (the Clinical Dementia Rating Scale in its Sum of Boxes) (Pinto et al. 2019). However, obtaining biochemical indicators is difficult and invasive, and scale tests can be easily disrupted by non-pathological subjective factors. Additionally, MCI exhibits heterogeneity in cognitive function and clinical progression, which makes prognostic outcomes difficult to predict. Current imaging tools for diagnosing and classifying MCI and AD mainly include MRI and PET. MRI can identify brain structural and functional changes caused by AD at different stages (such as hippocampal volume, cortical thickness) (Hashemi et al. 2023); 18F-FDG PET evaluates brain metabolism by measuring local glucose consumption in brain tissue (Qiu et al. 2022). Recent research shows that, in the MCI phase, 18F-FDG PET may show features of neurodegeneration earlier than MRI (Kang et al. 2021; Cahill and Huang 2017). As the disease progresses, a characteristic and progressive reduction in the glucose metabolic rate can be observed in specific regions, suggesting that 18F-FDG PET may better predict the transition from MCI to AD than MRI (Abe et al. 2020).

Radiomics is a fast machine learning based analysis paradigm focused on interrogating disease heterogeneity through image features (Kumar et al. 2012). Initially, radiomics was widely used in oncology (Thawani et al. 2018), and recently being utilized for diagnosing and classifying degenerative neurological diseases (Salvatore et al. 2021). Ranjbar and others divided patients into AD, MCI, and control groups, and compared radiomic texture features within the hippocampal region, showing excellent performance in distinguishing AD and control groups (89% AUC) as well as MCI and control groups (86% AUC) (Ranjbar et al. 2019). A new method proposed by Nanni and others combined the texture descriptors of the AD brain with voxel-based features, demonstrating optimal classification performance in line with the latest research (94% AUC for classifying AD vs. control, 70% for distinguishing between sMCI and pMCI) (Nanni et al. 2019). However, these studies were based on MRI image analysis and did not involve whole brain regions (Salmanpour et al. 2021). They mainly focused on brain structure analysis or traditionally defined areas such as the hippocampus (Sun et al. 2018), and did not explore the heterogeneity of glucose metabolism in other brain regions. Therefore, in this study, our aim was to develop a radiomics paradigm of whole brain PET images to analyze brain regions associated with the progression of MCI and explore disease progression pattern.

## Materials and methods

### Patient selection

**Fig. 1:**
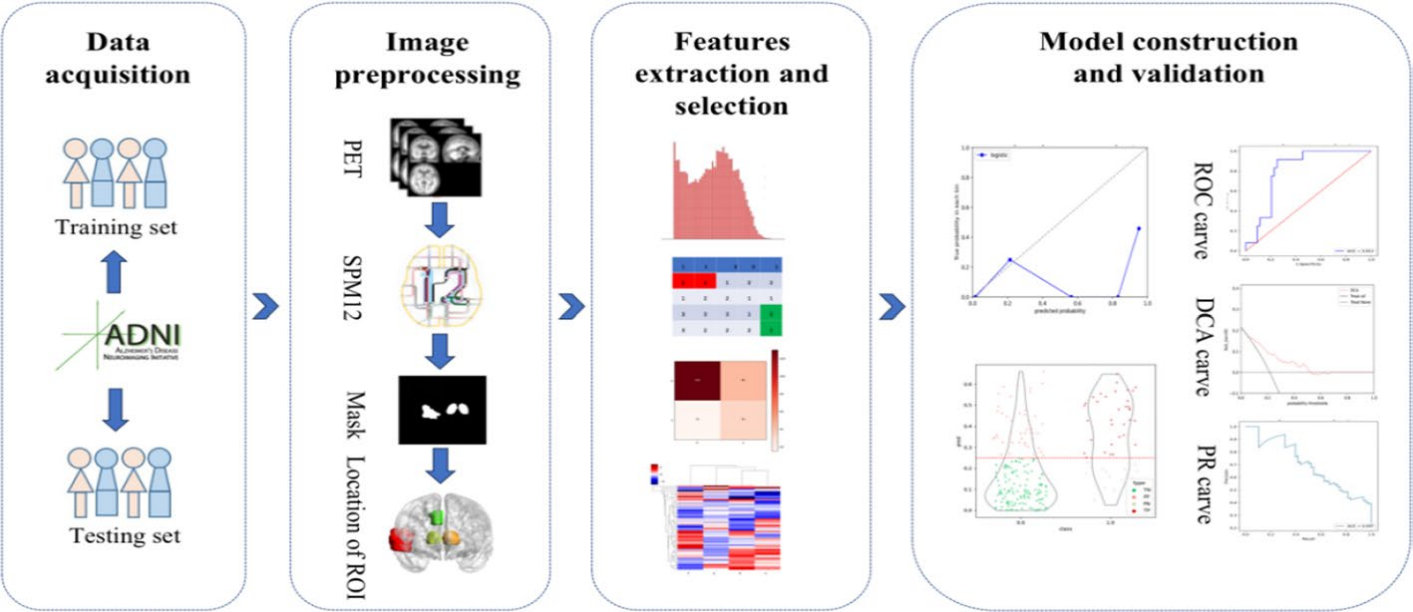
Research flow chart

This study utilized data from the Alzheimer's disease neuroimaging initiative (ADNI) project, specifically ADNI2 cohorts, which include 3 years of follow-up records. Ethics committee approval was not required as the data were sourced from a publicly available database (adni.loni.usc.edu). We analyzed 278 MCI patients, incorporating demographic information such as age, gender, weight, and educational background. Patients were classified as progressive MCI (pMCI, n = 60) if they were diagnosed with AD within 48 months, or as stable MCI (sMCI, n = 218) if the diagnosis remained unchanged. For voxel-based analysis, 30 patients from each group were randomly selected. The cohort was also divided into a training set (n = 222) and a validation set (n = 56) at an 8:2 ratio for model training and validation, respectively. The study's workflow is depicted in Fig. 1.

### Image preprocessing

18F-FDG PET images were processed as outlined in the ADNI project's online materials (https://adni.loni.usc.edu/methods/pet-analysis-method/pet-analysis/). Using SPM12 software (Wellcome Department of Imaging Neuroscience, Institute of Neurology, London, United Kingdom) on Matlab2016b (Mathworks Inc, Sherborn, MA, United Kingdom), images were standardized to Montreal Neurological Institute (MNI) space and smoothed with an 8 mm Gaussian filter. The normalized images were then rescaled to a 0–255 range to normalize FDG uptake values across subjects.

### Identification of ROIs related to MCI progression

Using preprocessed images from both patient groups, voxel-wise two-sample t-tests were conducted to identify regions of interest (ROIs) associated with MCI progression. An FDR-corrected threshold of *P* ≤ 0.01 and a minimum cluster size of > 50 voxels were applied to define significant ROIs. These were mapped to the AAL (Anatomical Automatic Labeling) brain atlas, identifying four key ROIs (ROI1-4) for further analysis.

### Feature extraction and analysis

Radiomics features were extracted using the 3D Slicer tool (Fedorov A., Beichel R., Kalpathy-Cramer J., Finet J., Fillion-Robin J-C., Pujol S., Bauer C., Jennings D., Fennessy F.M., Sonka M., Buatti J., Aylward S.R., Miller J.V., Pieper S., Kikinis R. 3D Slicer as an Image Computing Platform for the Quantitative Imaging Network. Magnetic Resonance Imaging. 2012 Nov;30(9):1323–41. PMID: 22770690. PMCID: PMC3466397. Available at: https://www.slicer.org/), with each target ROI analyzed separately. A comprehensive set of 837 features per ROI was analyzed, encompassing grayscale, texture, shape, and wavelet-transformed features. The extracted radiomic features comply with the feature definitions described by the Imaging Biomarker Standardization Initiative (IBSI).

To standardize feature scales, Z-score normalization was applied. Feature dimension reduction was conducted through variance (threshold set at *P* < 0.3) and correlation analyses, followed by univariate logistic (*P* < 0.05) regression and gradient boosting decision tree (GBDT) analysis to select the most significant features for MCI progression.

### Model construction and evaluation

Logistic regression analysis was utilized to construct five predictive radiomics models based on individual and combined ROIs (Model 1–4 for individual ROIs and Model 5 for the combined ROI_total). ROC curve and confusion matrix analyses were employed to evaluate model performance, complemented by decision curve analysis (DCA) to assess clinical utility.

### Statistical analysis

Statistical analyses were performed using SPSS 20.0 (The SPSSAU project (2020). SPSSAU. (Version 20.0) [Online Application Software]. Retrieved from https://www.spssau.com) and R4.0 software (R Core Team (2020). R: A language and environment for statistical computing. R Foundation for Statistical Computing, Vienna, Austria. URL https://www.R-project.org/.). The normality of variables was assessed with the Kolmogorov–Smirnov test. Depending on the distribution, the Student’s t-test or Mann–Whitney U test was applied for quantitative variables, while chi-square tests were used for qualitative variables. A *p* value < 0.05 was deemed statistically significant. ROC curve analysis was conducted for each model to calculate metrics like AUC, accuracy, precision, and recall, thereby assessing diagnostic accuracy.

## Result

### Data analysis

Demographic characteristics of the study participants are detailed in Table 1. Comparative analysis revealed no statistically significant differences in demographic parameters between the training and validation groups (*p* > 0.05), indicating a homogeneous distribution across both cohorts. Furthermore, evaluations of age, gender, weight, and educational background showed no significant disparities between the pMCI and sMCI groups within both the training and validation cohorts.

**Table 1:** Characteristics of MCI patients in the training cohort and validation cohort

Characteristics	Training (n = 222)		*P* value	Validation (n = 56)		*P* value
	sMCI (n = 174)	pMCI (n = 48)		sMCI (n = 44)	pMCI (n = 12)	
Demographic information, mean (SD), years, kilogram						
Age	75 (7.79)	77.31 (6.75)	0.31	72.68 (5.71)	78.42 (6.26)	0.57
Sex (M/F)	94/80	31/17	0.2	36/8	8/4	0.1
Education year	16.01 (2.84)	16.23 (2.20)	0.62	15.82 (2.38)	16.53 (2.84)	0.35
Weight	77.32 (14.34)	76.41 (15.80)	0.71	84.06 (15.58)	80.66 (17.45)	0.52

*P* > 0.05

*MCI* mild cognitive impairment

### Localization of brain regions with glucose metabolic differences related to MCI to AD progression

**Fig. 2:**
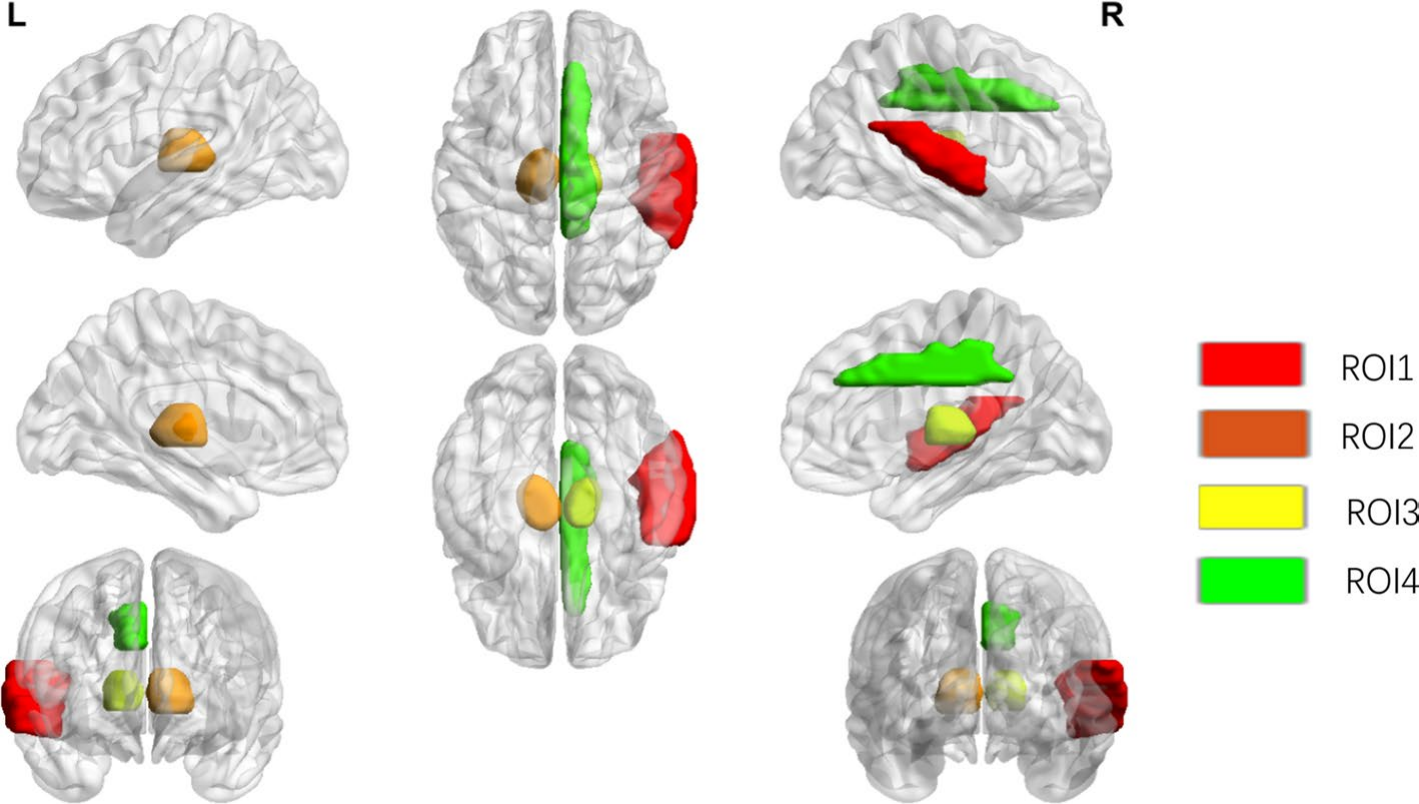
Results of t-test for two samples undergoing brain 18F-FDG positron emission computed tomography to assess metabolic differences in brain regions between sMCI patients and pMCI

Figure 2 illustrates the outcomes of voxel-based two-sample t-tests conducted on the training group, which comprised 218 stable MCI (sMCI) subjects and 60 progressive MCI (pMCI) subjects. The anatomical details of the identified target brain regions, which are implicated in the progression from MCI to AD, are detailed in Table 2. Four regions exhibited significantly reduced glucose metabolism, indicative of their involvement in disease progression. Specifically, ROI1 is situated in the Temporal lobe, suggesting its crucial role. Both ROI2 and ROI3 are located within the Thalamus, while ROI4 is found in the Limbic region, highlighting these areas as key sites of metabolic reduction associated with MCI advancement towards AD.

**Table 2:** Brain regions with significant differences between sMCI and pMCI in the ADNI cohort

Name	MNI coordinates	Cluster location (standardized automated anatomical labeling template)	Hemisphere
ROI1	61, − 40, 11	Right Cerebrum; Temporal Lobe; Superior Temporal Gyrus; Gray Matter	Right
ROI2	− 8, − 12, 8	Left Cerebrum; Sub-lobar; Thalamus; Gray Matter, Medial Dorsal Nucleus	Left
ROI3	16, − 24, 7	Right Cerebrum; Sub-lobar; Thalamus; Gray Matter; Pulvinar	Right
ROI4	5, 27, 29	Right Cerebrum; Limbic Lobe; Cingulate Gyrus; Gray Matter	Right

### Construction and evaluation of single ROI radiomics model

**Fig. 3:**
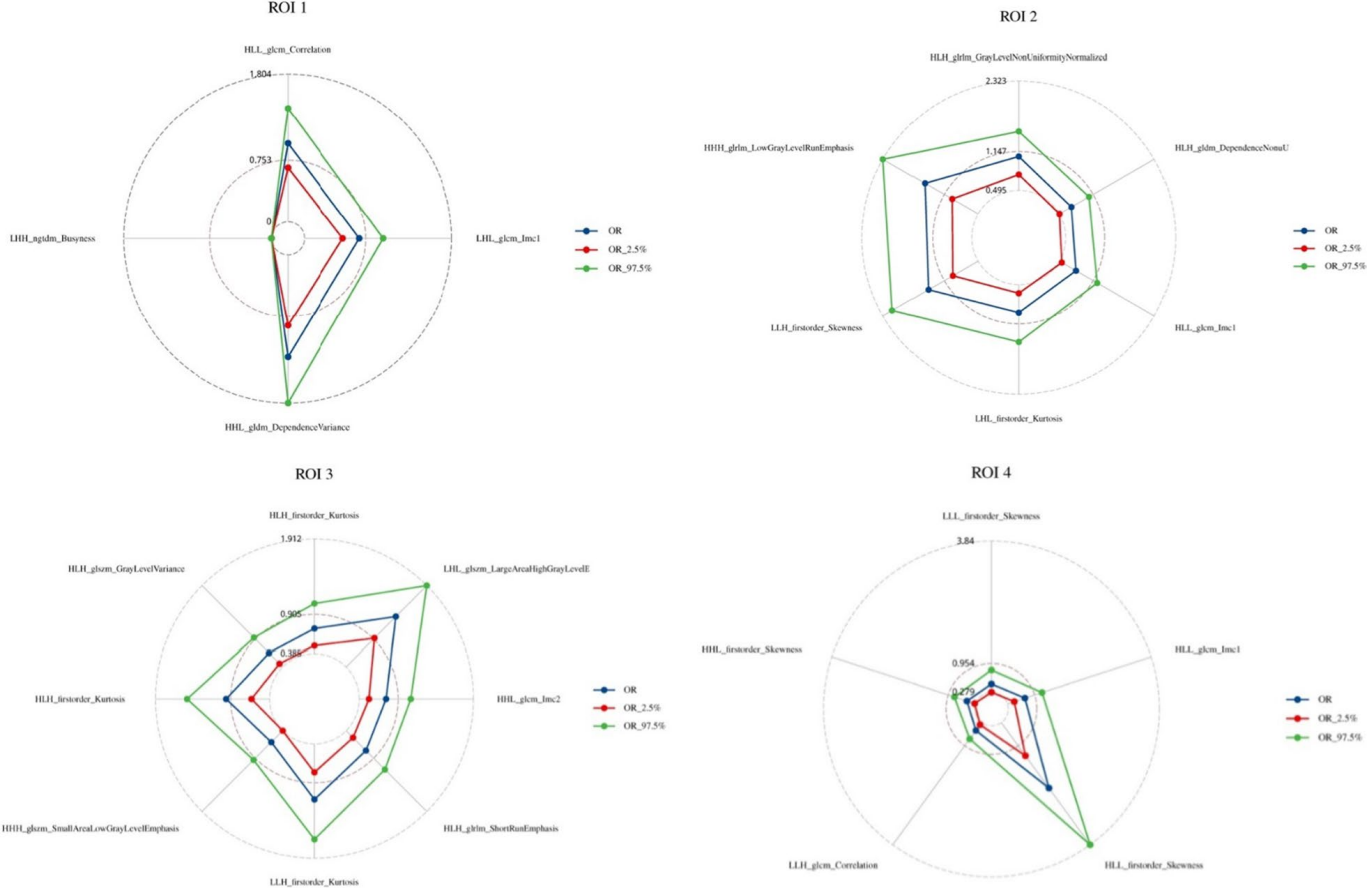
OR values and 95% confidence intervals for key imaging histological features in logistic models for each single brain region

Upon refining and selecting from the initial 837 radiomic features for each identified target ROI within the training group, we retained a specific number of key features for each region: 4 for ROI1, 6 for ROI2, 8 for ROI3, and 5 for ROI4. These selected features were used to construct the respective single ROI radiomics models (detailed in Additional file 1: Table S1). Figure 3 presents the odds ratios (ORs) and 95% confidence intervals for the key radiomic features across each model, illustrating their predictive relevance. The performance metrics for these models are consolidated in Table 3. Notably, the model leveraging features from ROI4 emerged as the most predictive: in the training dataset, it achieved an area under the curve (AUC) of 0.803 (95% CI 0.736, 0.865), with an accuracy of 75.4%, sensitivity of 72.9%, and specificity of 76.4%. For the validation dataset, this model showed an AUC of 0.733 (95% CI 0.559, 0.893), accuracy of 69.6%, sensitivity of 66.7%, and specificity of 70.5%. In contrast, the model based on ROI3 displayed the least favorable performance, with training set AUC at 0.75 (95% CI 0.685, 0.809) and a notably lower validation set AUC of 0.506 (95% CI 0.319, 0.693). Models for ROI1 and ROI2 demonstrated intermediate performance levels; ROI1's model had AUCs of 0.791 (95% CI 0.731, 0.846) in the training set and 0.812 (95% CI 0.707, 0.898) in the validation set, while ROI2’s model showed training and validation set AUCs of 0.741 (95% CI 0.672, 0.81) and 0.646 (95% CI 0.511, 0.777), respectively. Detailed confusion matrices for each model are provided in Additional file 1: Figs. S1 and S2, further illustrating their diagnostic accuracy.

**Table 3:** Diagnostic performance evaluation metrics of single brain region (ROI1, ROI2, ROI3, ROI4) versus multiple brain region (ROItotal) imaging histological models

Model	AUC		Accuracy		Sensitivity		Specificity		Positive prediction		Negative prediction		F1_score	
	Training	Validation	Training	Validation	Training	Validation	Training	Validation	Training	Validation	Training	Validation	Training	Validation
ROI1	0.791 (0.731, 0.846)	0.812 (0.707, 0.898)	0.734	0.625	0.688	0.917	0.688	0.917	0.429	0.355	0.897	0.96	0.528	0.512
ROI2	0.741 (0.672, 0.81)	0.646 (0.511, 0.777)	0.658	0.607	0.771	0.583	0.771	0.583	0.363	0.292	0.908	0.844	0.493	0.389
ROI3	0.75 (0.685, 0.809)	0.506 (0.319, 0.693)	0.586	0.464	0.875	0.5	0.2	0.875	0.328	0.2	0.936	0.769	0.477	0.286
ROI4	0.803 (0.736, 0.865)	0.733 (0.559, 0.893)	0.757	0.696	0.729	0.667	0.729	0.667	0.461	0.381	0.911	0.886	0.565	0.485
ROI_total	0.884 (0.845, 0.921)	0.816 (0.705, 0.909)	0.73	0.625	0.917	1.0	0.917	1.0	0.44	0.364	0.967	0.4	0.595	0.533

### Construction and evaluation of multiple ROIs model

**Fig. 4:**
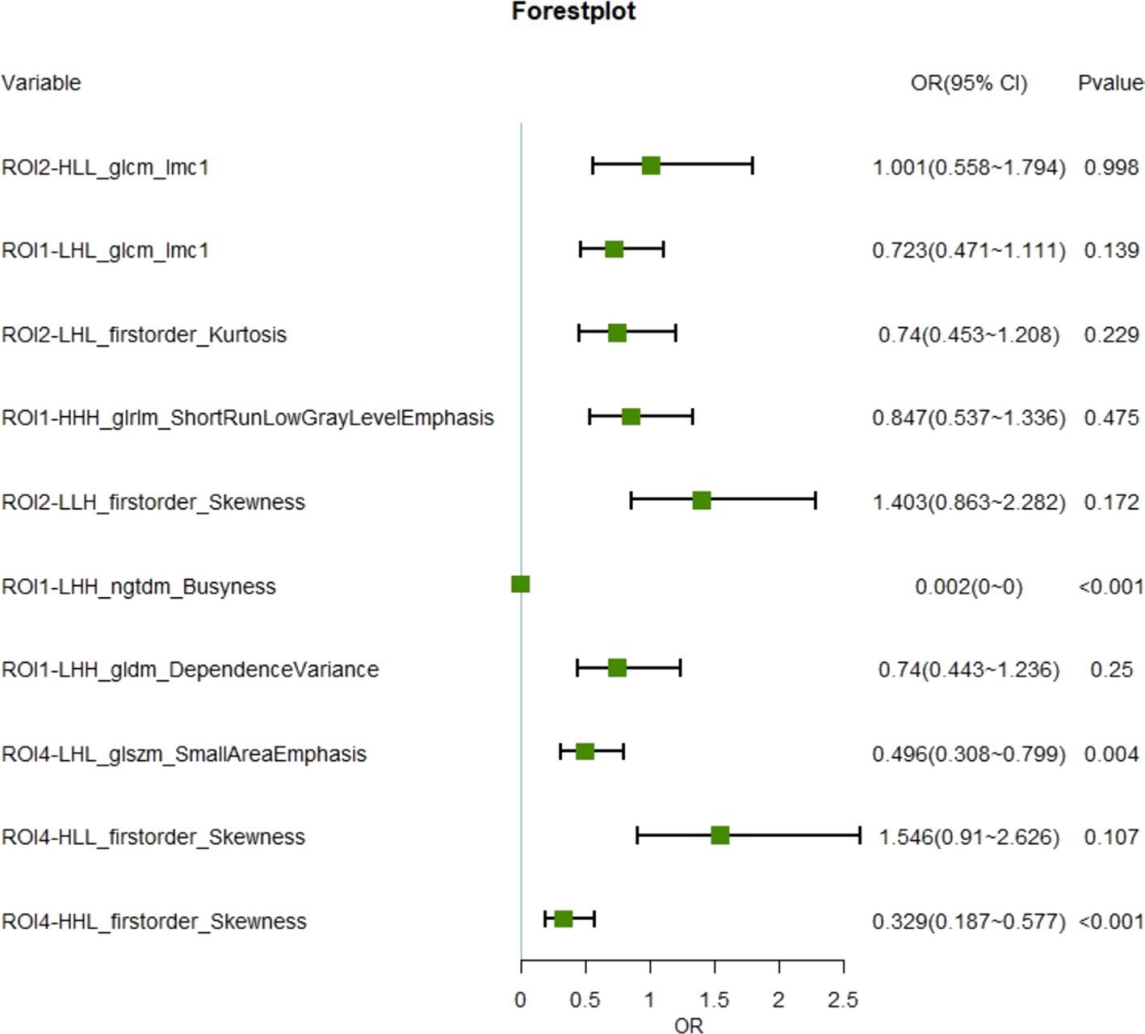
Forest plot of ratio ratios for different predictors. The multi-brain area radiomics model consists of 10 key features

**Fig. 5:**
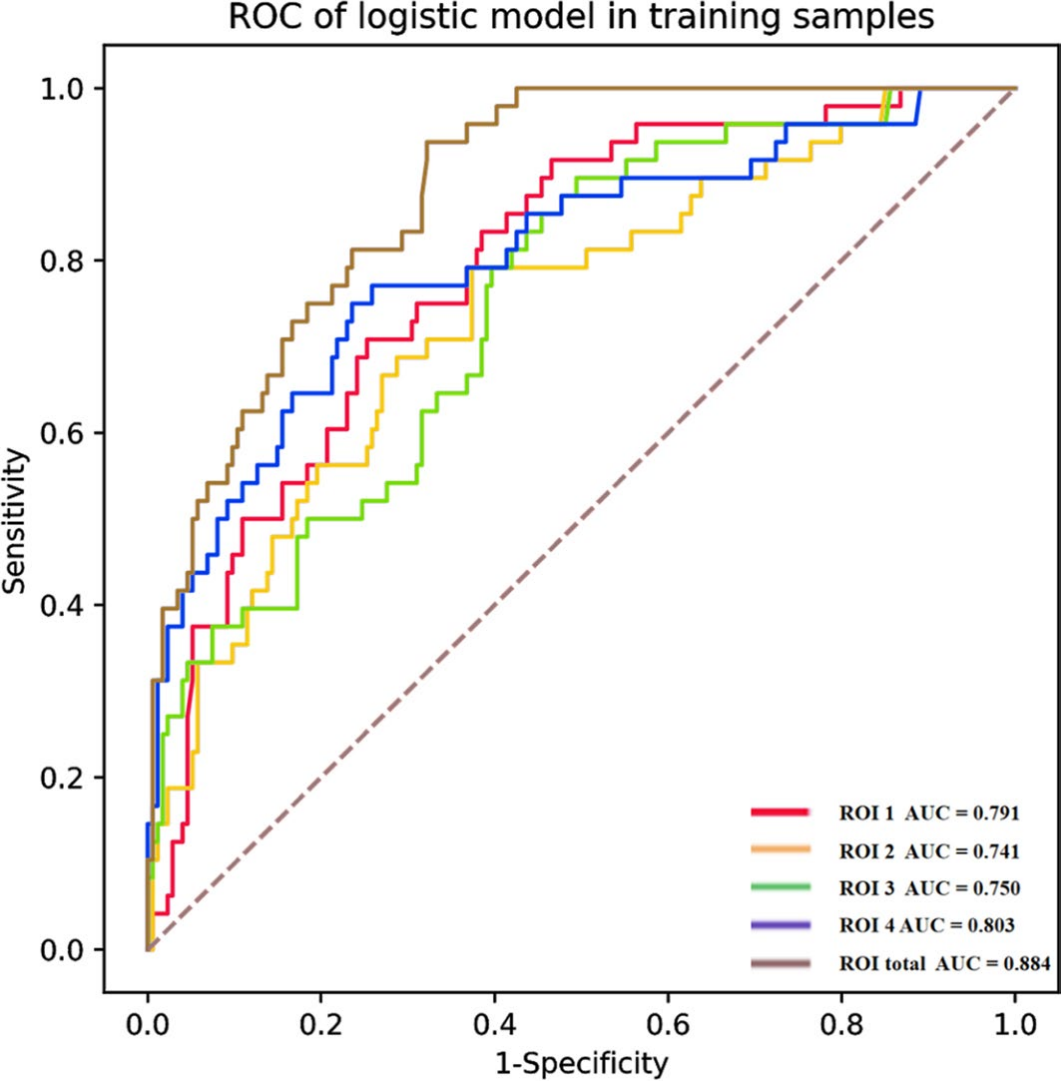
The ROC curves of the ROItotal prediction model in the training group group

**Fig. 6:**
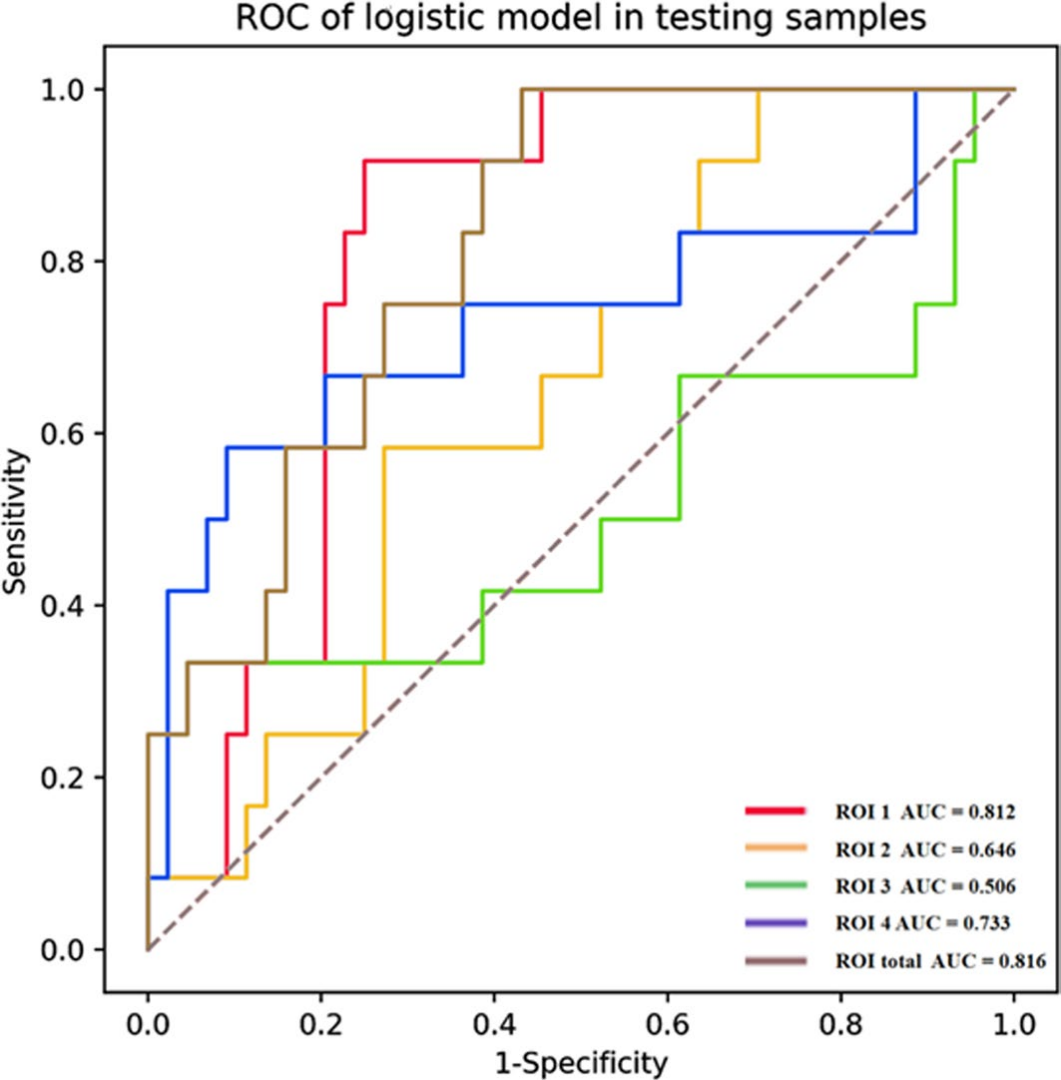
The ROC curves of the ROItotal prediction model in the validation group

The integration of radiomic features from all identified regions resulted in the retention of 11 key features for the comprehensive multiple ROIs model (ROI_total). This model included 4 features from ROI1, 4 from ROI2, and 3 from ROI4, as detailed in Additional file 1: Table S1. The logistic regression analysis and the predictive significance of these features are illustrated in Fig. 4 through a forest plot. The performance of the ROI_total model is detailed in Table 3 and marks a significant enhancement over the individual ROI models. For the training set, the area under the curve (AUC) for the ROI_total reached 0.884 (95% CI 0.845, 0.921), and for the validation set, it achieved an AUC of 0.816 (95% CI 0.705, 0.909), making it the most predictive model among those evaluated (as depicted in Figs. 5, 6). The superior performance underscores the advantages of a holistic approach to radiomic feature integration. Further validating its clinical utility, the decision curve analysis (DCA) for the multi-region model is provided in Additional file 1: Fig. S5. Additionally, the precision–recall (PR) curve, along with its area under the curve, further emphasizes the model's diagnostic accuracy. The training set's PR-AUC was 0.697, and the validation set's was 0.587, as shown in Additional file 1: Figs. S3 and S4. These metrics highlight the ROI_total model's enhanced predictive capability and its potential impact on identifying MCI patients at risk of progressing to AD .

## Discussion

Our study introduces a sophisticated radiomics workflow utilizing 18F-FDG PET imaging to forecast the progression from MCI to AD. Initially, we focused on analyzing 18F-FDG images to pinpoint brain regions where glucose metabolism differs significantly during MCI transition. This analysis facilitated the extraction of a vast array of radiomic features from these critical areas, leading to the identification of pivotal imaging biomarkers. Subsequent model construction for individual brain regions highlighted the logistic model based on ROI4 (Limbic region) as the standout performer. Advancing our analysis, we integrated features from all identified regions through feature fusion, which markedly enhanced the predictive accuracy of our comprehensive multi-regional radiomic model over any single-region approach. These findings underscore the potential of radiomic analysis, specifically targeting areas with altered glucose metabolism, to longitudinally predict MCI conversion. This approach offers promising avenues for the non-invasive monitoring and clinical assessment of MCI, paving the way for early and targeted interventions.

AD evolves through pathological transformations in various brain regions, with MCI marking its incipient phase. Despite some MCI individuals progressing to AD, the full spectrum of cognitive symptoms often remains latent, complicating clinical detection (Yang et al. 2012). Differentiating between MCI patients on the verge of progressing to AD (pMCI) and those likely to maintain stability (sMCI) is crucial for initiating timely interventions. Our investigation identified significant metabolic disparities between pMCI and sMCI predominantly in the right superior temporal gyrus, Thalamus, and Limbic Lobe, aligning with prior research. These disparities suggest that impairments in these areas could disrupt cognitive functions and language networks, mirroring MCI's clinical presentations and offering insights into MCI's evolution towards AD and the exploration of targeted treatments. The Superior Temporal Gyrus (STG), acting as the auditory language hub, is crucial for encoding situational memory, processing speech, and comprehending language (Ding et al. 2014; Susanto et al. 2015). Damage to the STG, often susceptible in early MCI and AD stages, may lead to receptive aphasia, impacting the language network (Karas et al. 2004; Dobricic et al. 2022). This damage could underlie the linguistic challenges observed in MCI patients. The Limbic Lobe (LL) plays a pivotal role in language perception, processing, and semantic interpretation (Graff-Guerrero et al. 2005). Our findings suggest that pMCI individuals exhibit diminished functional activity in the LL, potentially exacerbating cognitive decline as MCI advances to AD (Li et al. 2016). This observation is consistent with prior studies, reinforcing the link between MCI and language dysfunction. Furthermore, the Thalamus, integral to the regulation of basal ganglia and cortical neurons, influences the basal ganglia-thalamic-cortical circuit. Degeneration in the Thalamus could thus precipitate both motor and cognitive impairments. Its connection to the hippocampus, essential for situational memory, implies that Thalamic atrophy, particularly in its anterior regions, might indirectly compromise hippocampal functionality, contributing to memory deficits. This relationship provides a rationale for the observed correlation between Thalamic damage and MCI progression, emphasizing the need for further research into the neurobiological underpinnings of MCI and AD.

Biomarkers are indispensable for the early detection and prognostication of Alzheimer's disease (AD), with neuropsychological, biochemical, and neuroimaging markers being among the most extensively studied (Blennow and Zetterberg 2018; Genin et al. 2011). Neuroimaging analysis traditionally focuses on structural features like hippocampal volume and cortical thickness (Talwar et al. 2021; Zhou et al. 2021). Interestingly, research indicates that 18F-FDG PET imaging can reveal neurodegenerative patterns at the MCI stage sooner than MRI, suggesting a decline in glucose metabolism in certain brain regions as a potential early indicator of AD progression (Prem et al. 2022; Pagani et al. 2017). Our study leverages PET radiomics, achieving predictive model AUC scores of 0.884 and 0.816 in training and validation sets, respectively. These results surpass those of models based on MRI radiomics, such as the one developed by Shu et al. (2021), which reported lower AUC values of 0.722 and 0.692. This underscores the potential of PET radiomics for enhancing early AD prediction accuracy. Following the establishment of ROI templates through SPM analysis, we meticulously extracted features from these predefined regions. Unlike conventional radiomics, which often includes shape features derived from manually segmented ROIs of varied geometries, our analysis omitted shape features due to the standardized nature of our ROI templates. This approach minimized individual differences in brain tissue shape through normalization and smoothing processes during image preprocessing, ensuring that our feature set focused on functional rather than structural discrepancies (Binczyk et al. 2021; Chetan and Gleeson 2021; Mayerhoefer et al. 2020).

In our analysis, the absence of ROI3 radiomics features in the key feature set for the ROI_total model, coupled with the lower predictive performance of the ROI3-based model, suggests a diminished correlation between the ROI3 location and the pathological evolution from MCI to AD. This inference is bolstered by research on the dorsal hypothalamic nucleus (DMH), a pivotal region influencing circadian rhythms, temperature regulation, and energy balance, among other functions. Given that DMH's impairment symptoms do not align closely with those of MCI or AD (van de Mortel et al. 2021; Carlson et al. 2020), it's plausible that its involvement in AD progression is minimal. Conversely, the ROI4 area, which we identified as highly predictive of AD progression, is crucial for memory functions and is well-documented in AD pathology. This is supported by findings from Feng et al., who identified hippocampal texture as a potential AD biomarker, underscoring the significance of imaging omics in distinguishing between AD, MCI, and cognitively normal individuals.

However, our study is not without limitations. The preprocessing steps for PET images, including smoothing, may obscure some texture details, potentially influencing the reliability of radiomic feature extraction. Additionally, our retrospective analysis, based on a public dataset, necessitates further validation through external datasets or prospective studies to enhance its generalizability. Another consideration is our method of dividing the study cohort into training and validation groups based on the sequence of entries into the ADNI database, introducing a potential bias due to evolving imaging technologies and methodologies over time. These limitations highlight areas for future research and underscore the need for continuous refinement in the methodology and validation of radiomic analyses in AD research.

## Conclusion

Our findings highlight the Limbic region (ROI4) as exhibiting the strongest correlation with the progression from MCI to AD, underscoring its pivotal role in the disease's advancement. Notably, the PET brain radiomics model incorporating multiple brain regions (ROI_total) surpasses single-region models in its ability to precisely identify MCI patients at a heightened risk of transitioning to AD. This comprehensive approach not only enhances predictive accuracy but also offers significant insights into the multifaceted nature of brain pathology in AD. Our study provides a valuable framework for non-invasive diagnostic techniques and supports the implementation of early, targeted intervention strategies in MCI management, potentially altering the clinical trajectory for individuals at risk of Alzheimer's.

### Supplementary Information

**Additional file 1: Evaluation of the model: Table S1.** Key feature subsets for ROI 1–4 and ROI total. **Fig. S1.** The distribution of Confusion matrix of each single brain region and multi brain region model in the training cohort from left to right is ROI 1–4 and ROI total. **Fig. S2.** The distribution of Confusion matrix of each single brain area and multi brain area model in the validation cohort from left to right is ROI 1–4 and ROI total. **Fig. S3.** The PR curve distribution of each single brain region and multi brain region prediction model in the training queue is ROI 1–4 and ROI total from left to right. **Fig. S4.** The distribution of PR curves in the validation queue for each single brain region and multi brain region prediction model is ROI 1–4 and ROI total from left to right. **Fig. S5.** The clinical curve decision curves of the training/testing groups for ROI 1–4 and ROI total models, respectively.

## Data Availability

The datasets generated and/or analyzed during the current research period can be obtained in the [ADNI] library, ADNI | ACCESS DATA (usc.edu).

## Abbreviations

MCI: Mild cognitive impairment
AD: Alzheimer's disease
sMCI: MCI not progressing to AD
PMCI: MCI progressing to AD
PET: Positron emission tomography
MRI: Magnetic resonance imaging
ROI: Region of interest
AUC: The area under curve
ROC: Receiver operator characteristic
AAL: Anatomical automatic labeling
DCA: Decision curve analysis
Pr: Percision–recall curves
