# Supplementary material for: Regional changes in brain metabolism during the progression of mild cognitive impairment: a longitudinal study based on radiomics
Source: EJNMMI Rep. 2024 Jul 1;8(1):19. doi: 10.1186/s41824-024-00206-8 (PMC11214937; doi:10.1186/s41824-024-00206-8)
Supplement: Supplementary file 1 — Additional file 1: Evaluation of the model: Table S1. Key feature subsets for ROI 1–4 and ROI total. Fig. S1. The distribution of Confusion matrix of each single brain region and multi brain region model in the training cohort from left to right is ROI 1–4 and ROI total. Fig. S2. The distribution of Confusion matrix of each single brain area and multi brain area model in the validation cohort from left to right is ROI 1–4 and ROI total. Fig. S3. The PR curve distribution of each single brain region and multi brain region prediction model in the training queue is ROI 1–4 and ROI total from left to right. Fig. S4. The distribution of PR curves in the validation queue for each single brain region and multi brain region prediction model is ROI 1–4 and ROI total from left to right. Fig. S5. The clinical curve decision curves of the training/testing groups for ROI 1–4 and ROI total models, respectively. [file 41824_2024_206_MOESM1_ESM.pdf]

TableS 1. Key feature subsets for ROI 1-4 and ROI total

group	Key Features	Number of features
ROI1	['ROI1_pet_wavelet-HLL_glcm_Correlation']<br>['ROI1_pet_wavelet-LHL_glcm_Imc1']<br>['ROI1_pet_wavelet-LHH_ngtdm_Busyness']<br>['ROI1_pet_wavelet-HHL_gldm_DependenceVariance']	4
ROI2	['ROI2_pet_wavelet-HLH_glrlm_GrayLevelNonUniformityNormalized']<br>['ROI2_pet_wavelet-HLH_gldm_DependenceNonUniformityNormalized']<br>['ROI2_pet_wavelet-HLL_glcm_Imc1']<br>['ROI2_pet_wavelet-LHL_firstorder_Kurtosis']<br>['ROI2_pet_wavelet-LLH_firstorder_Skewness']<br>['ROI2_pet_wavelet-HHH_glrlm_LowGrayLevelRunEmphasis']	6
ROI3	['ROI3_pet_wavelet-HLH_firstorder_Kurtosis']<br>['ROI3_pet_wavelet-LHL_glszm_LargeAreaHighGrayLevelEmphasis']<br>['ROI3_pet_wavelet-HHL_glcm_Imc2']<br>['ROI3_pet_wavelet-HLH_glrlm_ShortRunEmphasis']<br>['ROI3_pet_wavelet-LLH_firstorder_Kurtosis']<br>['ROI3_pet_wavelet-HHH_glszm_SmallAreaLowGrayLevelEmphasis']<br>['ROI3_pet_wavelet-LLL_firstorder_Kurtosis']<br>['ROI3_pet_wavelet-HLH_glszm_GrayLevelVariance']	8
ROI4	['ROI4_pet_wavelet-LLL_firstorder_Skewness']<br>['ROI4_pet_wavelet-HLL_glcm_Imc1']<br>['ROI4_pet_wavelet-HLL_firstorder_Skewness']<br>['ROI4_pet_wavelet-LLH_glcm_Correlation']<br>['ROI4_pet_wavelet-HHL_firstorder_Skewness']	5
ROItotal	['ROI2_pet_wavelet-HLL_glcm_Imc1']<br>['ROI1_pet_wavelet-LHL_glcm_Imc1']<br>['ROI2_pet_wavelet-LHL_firstorder_Kurtosis']<br>['ROI1_pet_wavelet-HHH_glrlm_ShortRunLowGrayLevelEmphasis']	10

	['ROI2_pet_wavelet-LLH_firstorder_Skewness']<br>['ROI1_pet_wavelet-LHH_ngtdm_Busyness']<br>['ROI1_pet_wavelet-LHH_gldm_DependenceVariance']<br>['ROI4_pet_wavelet-LHL_glszm_SmallAreaEmphasis']<br>['ROI4_pet_wavelet-HLL_firstorder_Skewness']<br>['ROI4_pet_wavelet-HHL_firstorder_Skewness']	

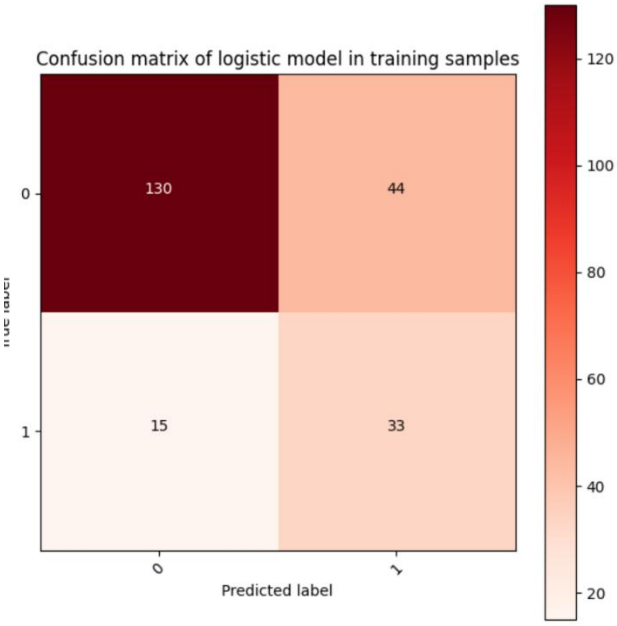

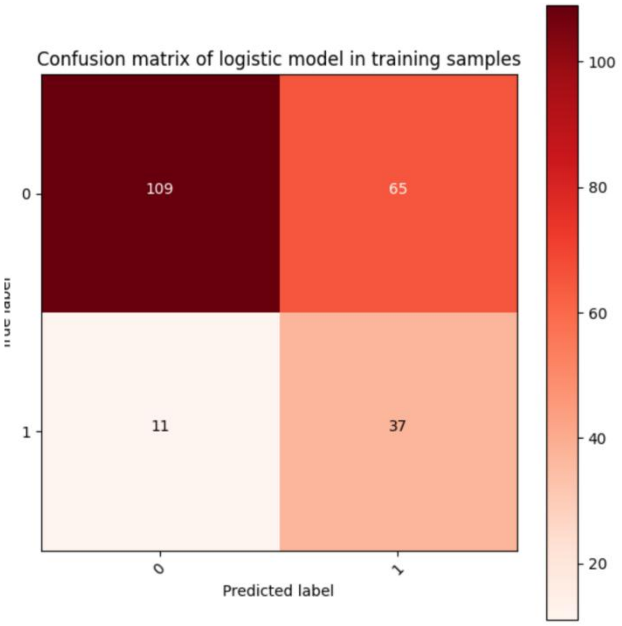

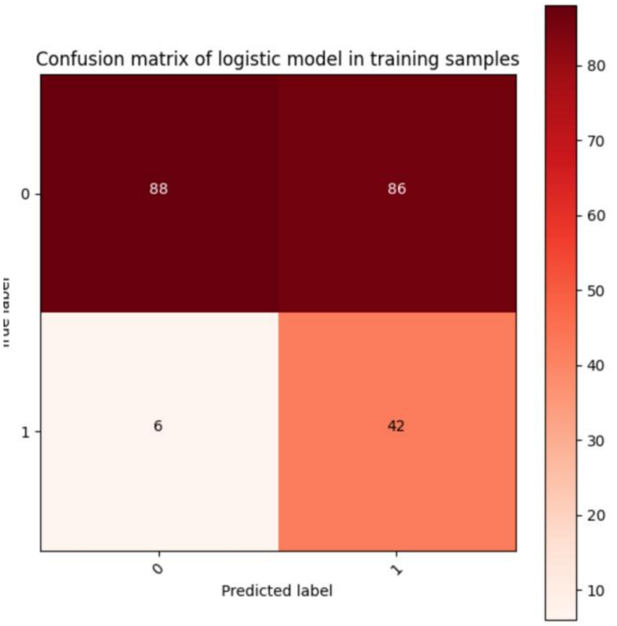

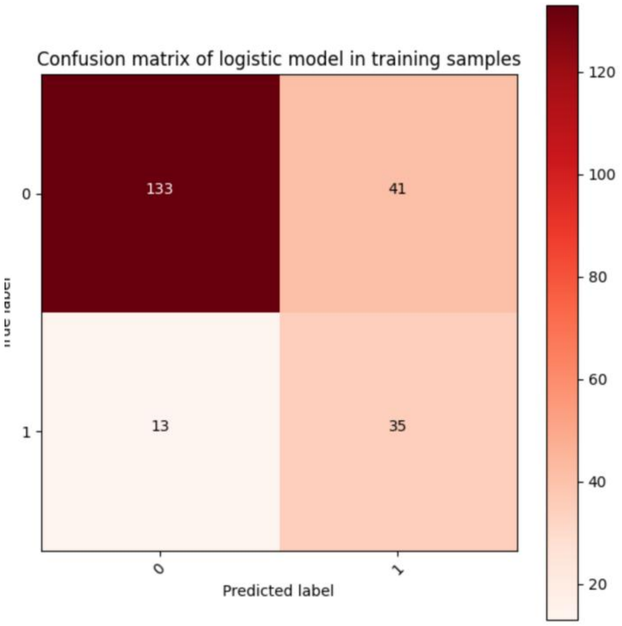

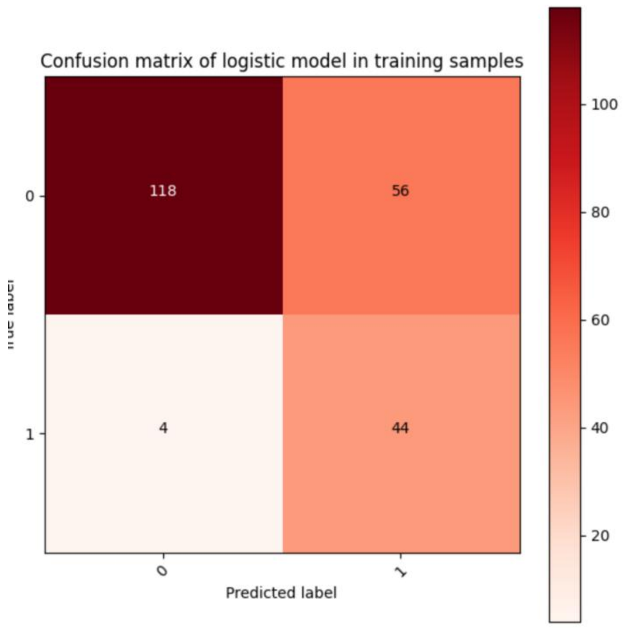

Figure S1.The distribution of Confusion matrix of each single brain region and multi brain region model in the training cohort from left to right is ROI 1-4 and ROI total.

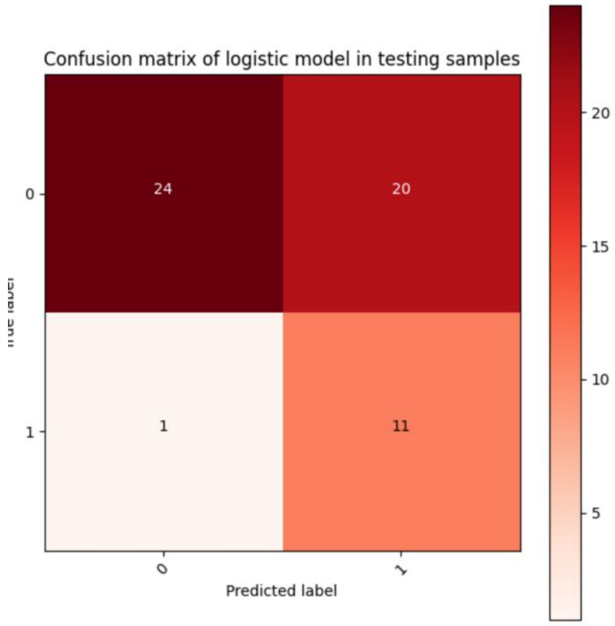
Confusion matrix of logistic model in testing samples
24
20
1
11
0
1
Predicted label
5
10
15
20

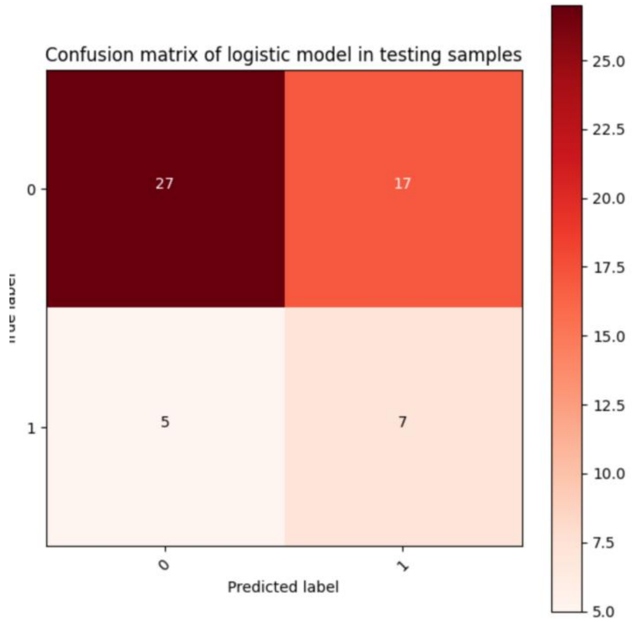
Confusion matrix of logistic model in testing samples
27
17
5
7
0
1
Predicted label
5.0
7.5
10.0
12.5
15.0
17.5
20.0
22.5
25.0

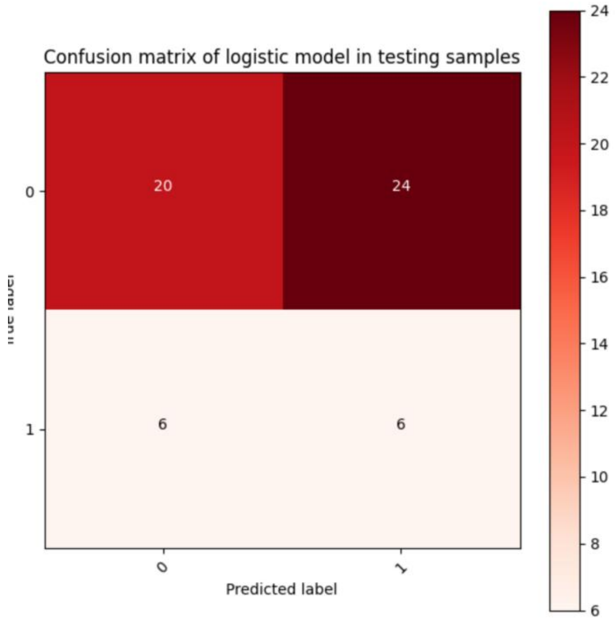
Confusion matrix of logistic model in testing samples
20
24
6
6
0
1
Predicted label
6
8
10
12
14
16
18
20
22
24

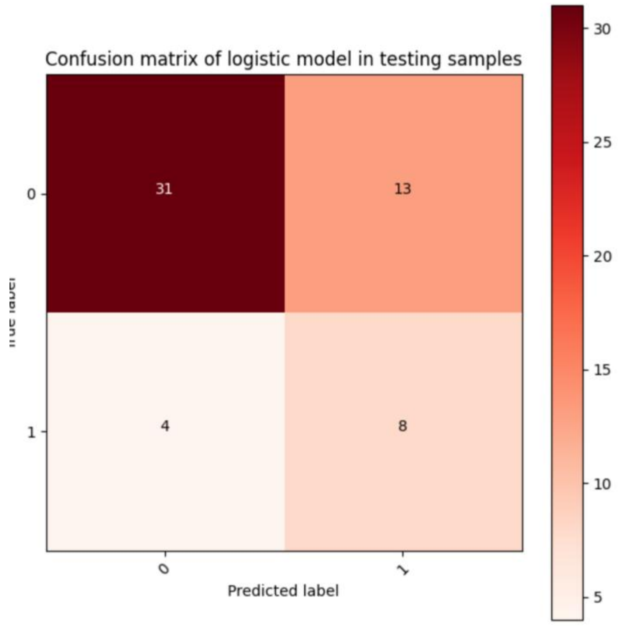
Confusion matrix of logistic model in testing samples
31
13
4
8
0
1
Predicted label
5
10
15
20
25
30

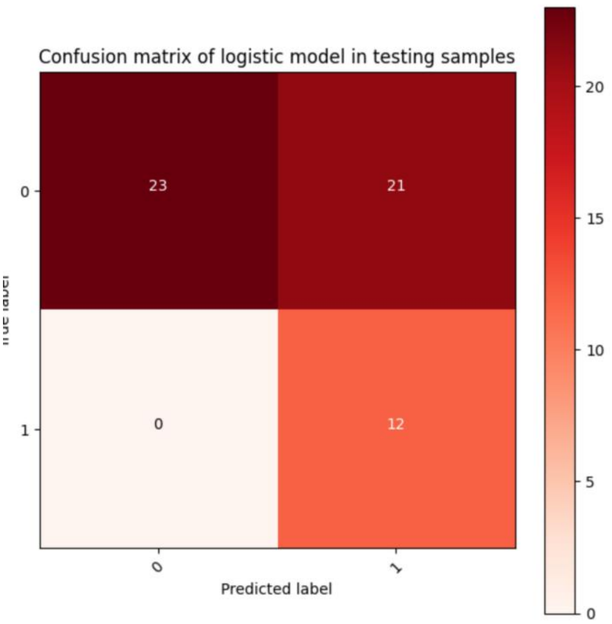

FigureS 2.The distribution of Confusion matrix of each single brain area and multi brain area model in the validation cohort from left to right is ROI 1-4 and ROI total.

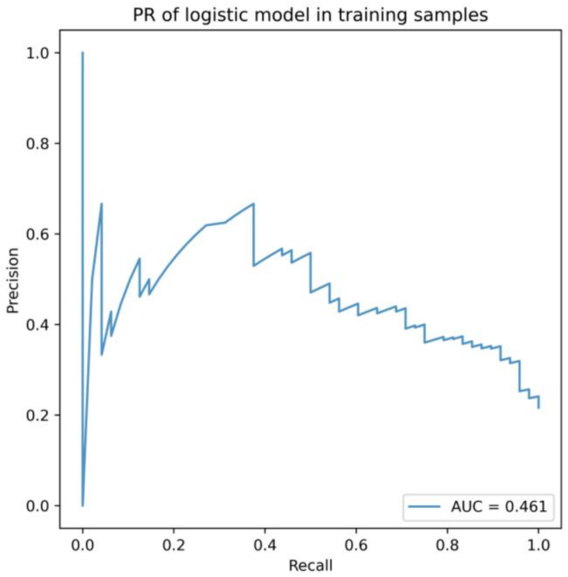

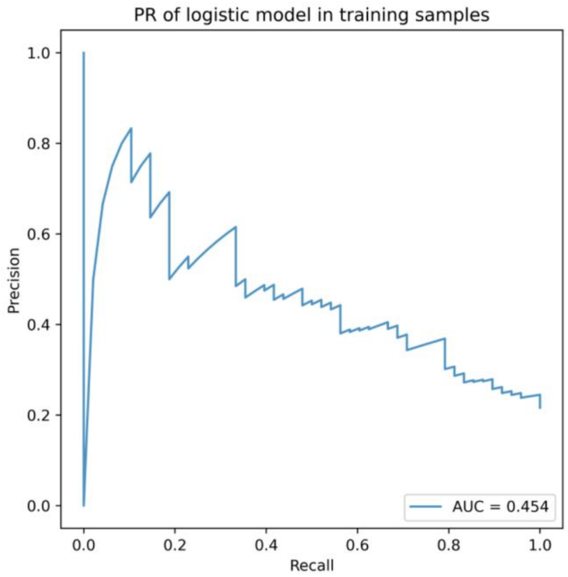

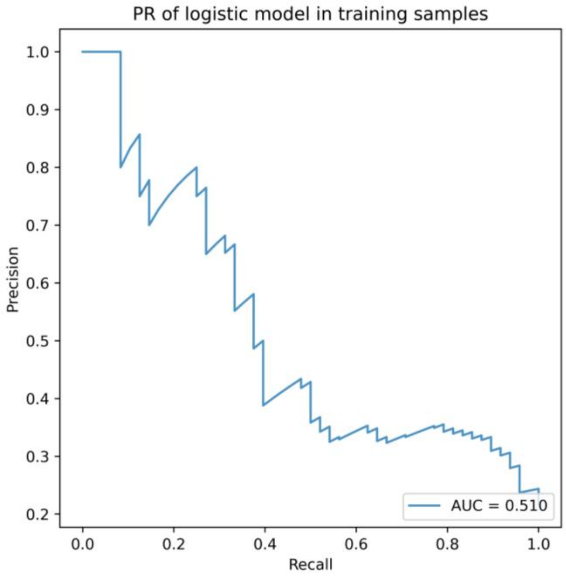

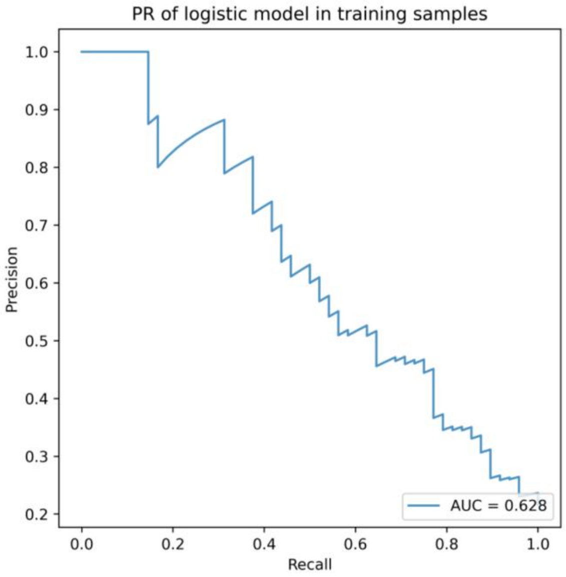

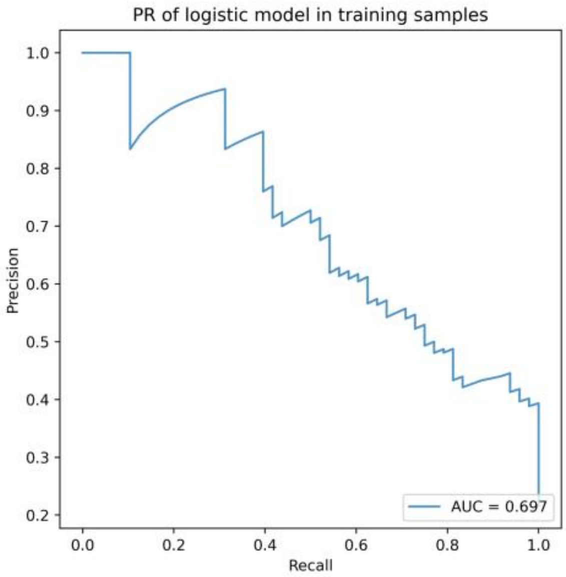

FigureS3.The PR curve distribution of each single brain region and multi brain region prediction model in the training queue is ROI 1-4 and ROI total from left to right.

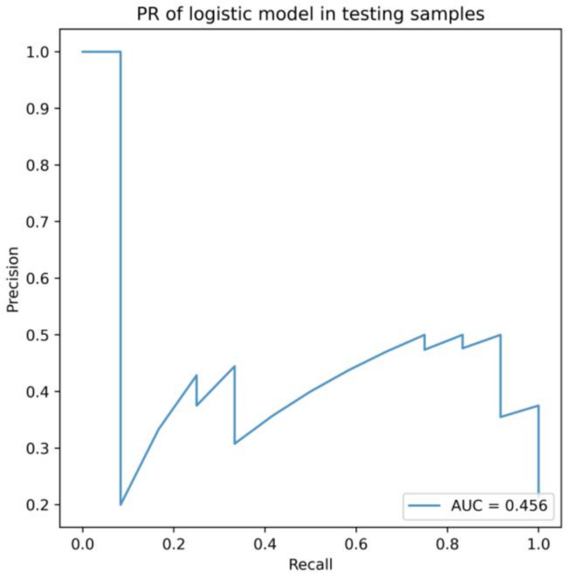
PR of logistic model in testing samples
Precision
Recall
AUC = 0.456

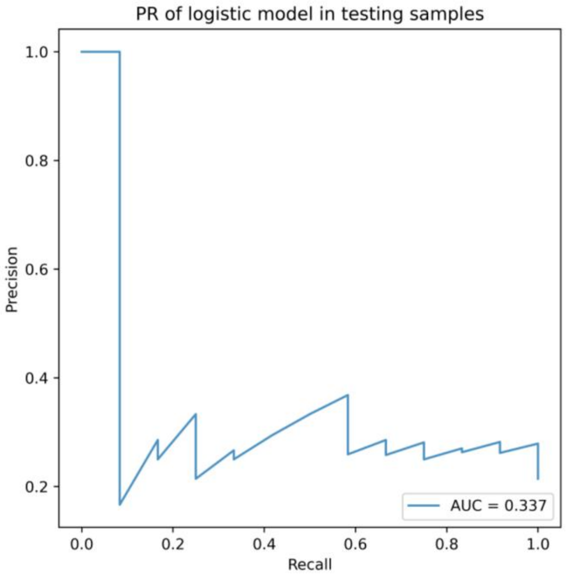
PR of logistic model in testing samples
Precision
Recall
AUC = 0.337

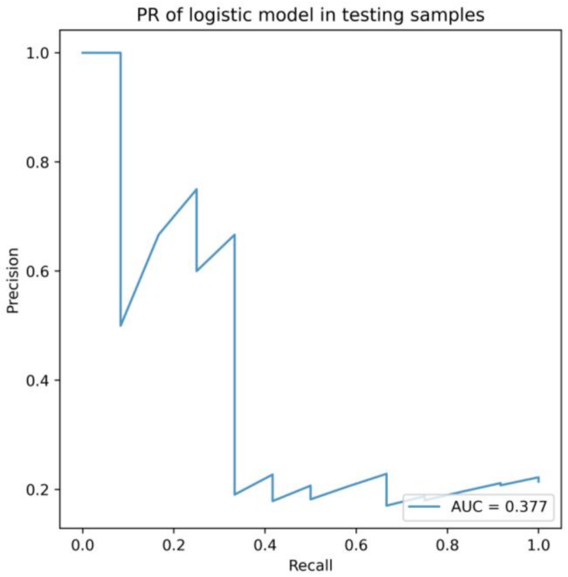
PR of logistic model in testing samples
Precision
Recall
AUC = 0.377

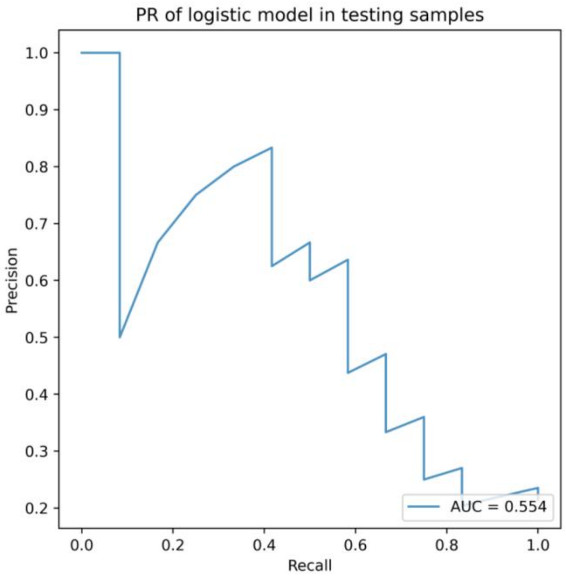
PR of logistic model in testing samples
Precision
Recall
AUC = 0.554

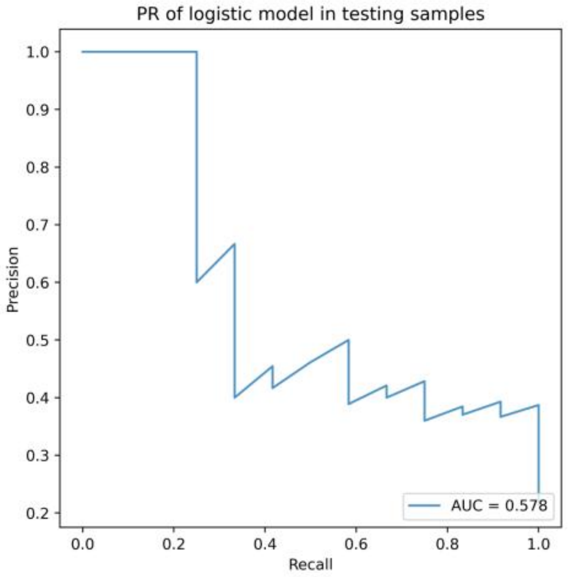

FigureS4.The distribution of PR curves in the validation queue for each single brain region and multi brain region prediction model is ROI 1-4 and ROI total from left to right

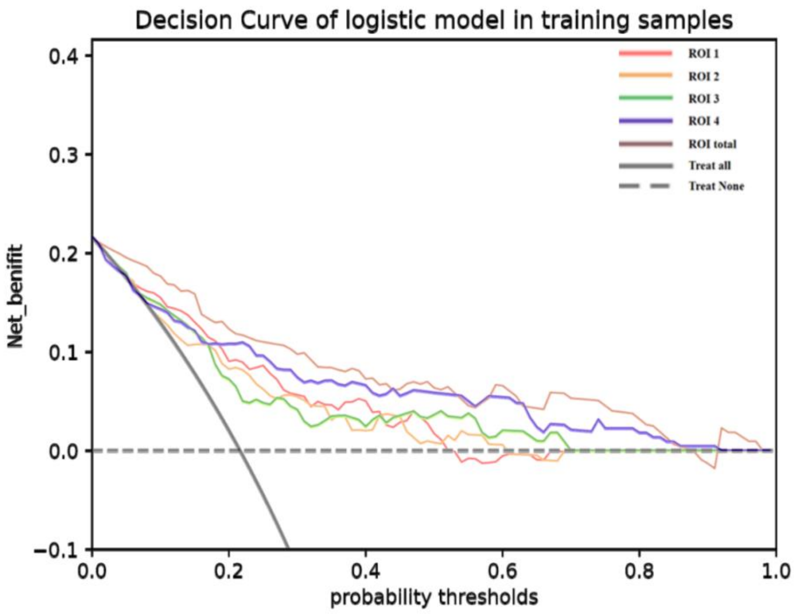

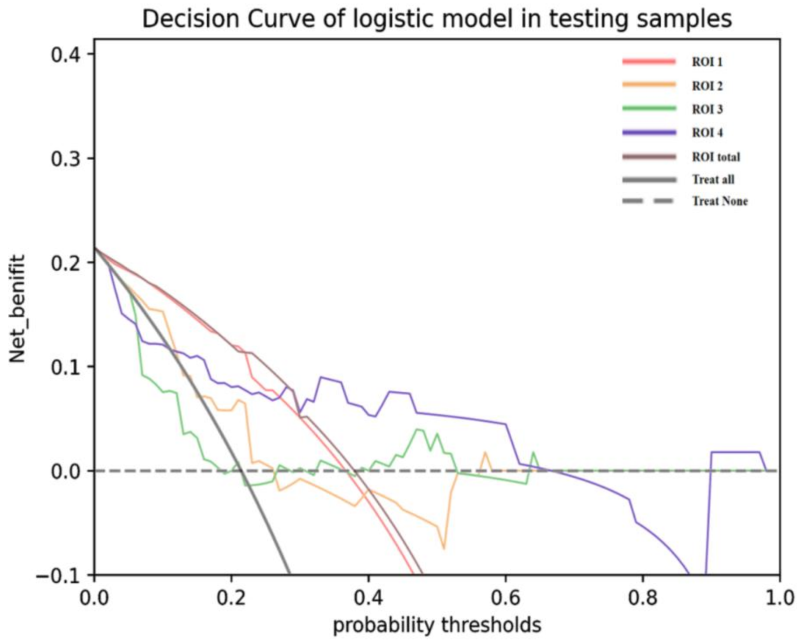

Figure S5. the clinical curve decision curves of the training/testing groups for ROI 1-4 and ROI total models, respectively.
